# Aortic valve replacement in a patient with self-reported systemic multiple metal allergy

**DOI:** 10.1007/s11748-021-01712-3

**Published:** 2021-09-25

**Authors:** Saori Nagura, Mari Sakai, Hayato Obi, Kazuaki Fukahara

**Affiliations:** 1 Department of Cardiovascular Surgery, Minaminagano Medical Center, Shinonoi General Hospital, 666-1, Shinonoi-Ai, Nagano, 388-8004 Japan; 2 First Department of Surgery, University of Toyama, Toyama, Japan

**Keywords:** Metal allergy, Aortic valve replacement, Aortic valve stenosis

## Abstract

We report our experience with aortic valve replacement in a patient with severe aortic stenosis who had self-reported allergies to multiple metals. The patient was a 74-year-old man. He developed palmoplantar pustulosis after using a dental prosthesis, and a patch test revealed reactions to several metals; therefore, he was diagnosed with systemic metal allergy. His condition progressed to severe aortic stenosis, and bioprosthetic aortic valve replacement was planned. The Avalus valve (Medtronic, Minneapolis, MN, USA) was selected for aortic valve replacement, since the patient reported having allergies to several metals. While most devices used in cardiac surgery contain some amount of metal, the Avalus bioprosthetic valve does not contain metal in the stent and has been extremely useful for aortic valve replacement in patients with suspected metal allergies.

## Introduction

In recent years, cardiac, dental, and orthopedic surgeries have frequently required the implantation of artificial devices in the body; however, there have been several reports of allergic reactions to various implanted devices [1–5, 9, 10]. Numerous devices and artificial organs used in cardiac surgery contain a certain amount of metal. We report our experience with aortic valve replacement (AVR) with the metal-free Avalus valve in a patient with severe aortic stenosis with self-reported allergy to multiple metals.

## Case

The patient was a 74-year-old man diagnosed with systemic metal allergy, due to palmoplantar pustulosis after using a dental prosthesis containing cobalt (Co), copper (Cu), and gold (Au) 20 years ago. The patient had severe aortic stenosis with an aortic valve area of 0.85 cm^2^. He exhibited dizziness and shortness of breath on exertion. Therefore, surgical intervention was deemed necessary. The patient stated that a patch test had been performed for the metal allergy 20 years ago, and several skin reactions with blisters occurred on the third day of the patch test. Only silver (Ag) and barium (Ba) were determined to be usable, and subsequent dental treatment was performed. The results could not be confirmed from the medical records at the time of diagnosis, because the retention period of the medical records had elapsed. Since the patient’s report during the diagnostic and examination process seemed to be specific and reliable, it was concluded that he was allergic to various metals, and therefore, metal-containing materials should be avoided for implants that are to remain in the body after cardiac surgery. We considered performing a patch test again before the surgery; however, we decided against the use of metal-containing materials for the implant that would remain in the body, and used a metal-free stented bioprosthetic valve, i.e., the Avalus valve (Medtronic, Minneapolis, MN, USA) instead, since the patient wished to avoid the test because of the strong skin symptoms at the time of the previous test.

Although the patient had been diagnosed with idiopathic interstitial pneumonia 7 years ago, preoperative respiratory function tests yielded normal results (%VC: 103.7% and FEV1.0%: 79.41%), while the KL-6 level was 286 U/mL, which was not sufficiently high to indicate a high degree of interstitial pneumonia at that time. However, the possibility of postoperative acute exacerbation should always be a concern, and we endeavored to simplify that the procedure as far as possible.

Surgery was performed using the median sternotomy approach. Cardiopulmonary bypass (CPB) was established with cannulation of the ascending aorta and bicaval venous cannulation; cardioplegic arrest was achieved in an antegrade fashion. Aortotomy was performed in the ascending aorta. The aortic valve was a tricuspid structure with highly calcified leaflets. A 21-mm Avalus valve was implanted in the supra-annular position with pledgeted mattress sutures. Since the patient had bradycardia at the time of weaning from CPB, a temporary pacemaker wire was placed in the right ventricle. Sternal closure was performed using #5 polyester braided sutures (ETHIBOND Excel polyester suture, Ethicon, Somerville, NJ, USA). The duration of the procedure, CPB, and aortic cross-clamping was 190, 102, and 65 min, respectively, all of which were completed without blood transfusion. The temporary pacemaker wire was removed on the second postoperative day (POD). The patient recovered uneventfully after surgery and was discharged on POD 17. Two years have elapsed since the surgery, and the prosthetic valve is functioning well. There was no abnormality in the healing of the sternum, and the patient has not experienced any skin or systemic symptoms since the procedure.

## Discussion

In addition to the fields of dentistry and orthopedics, cardiac surgery frequently requires the implantation of artificial devices in the body. However, most of these artificially implanted devices contain some kind of metal component, and there have been many reports of allergies caused by these metallic constituents [1–5]. There are two types of metal allergy: metal contact allergy, which causes inflammation at the site in direct contact with the metal, and systemic metal allergy, which causes sweaty eczema, palmoplantar pustulosis, lichen planus, nummular eczema, subacute prurigo, erythroderma, and pseudo-atopic dermatitis at remote sites at which the metal-containing substance is absorbed into the body. The first step in diagnosis is patch testing, but this method is associated with several false positives and false-negative results. The definitive diagnosis is based on exacerbation of the skin rash with increased intake and improvement in the skin rash with reduced intake. However, severe systemic metal allergies can occur in some cases. Although the patch test is simple, its sensitivity and specificity are merely 70–80% [6]. It is also reported that more than 10% of healthy people will test positive for some metal on a patch test [7, 8].

In a study of 58 patients diagnosed with nickel allergy after ASD closure and device removal, 21 patients (47%) reported that skin redness, malaise (82%), chest pain (78%), headache (73%), and palpitations (58%) appeared after device implantation [9]. A study that included patients who underwent total knee replacement reported that the percentage of patients with pain and functional decline was significantly higher and patient satisfaction was significantly lower in the group that exhibited a positive reaction to the metal content on a patch test [10]. Therefore, it is believed that there are several cases where the immune reaction to the implants could cause some kind of disorder around the implant or in the entire body, even in the absence of skin symptoms.

Studies conducted in the field of cardiac surgery have reported severe nickel allergy with repeated paravalvular leakage [1], and cardiac tamponade due to metal allergy to ASD closure devices [2] and sternal wires [3], which may be the result of inflammation caused by allergic reactions at the implantation site. Therefore, the possibility of an allergic reaction to the metal content of the implanted device should also be considered in cases of device failure, such as prosthetic valve failure or pericarditis.

However, it is not easy to remove the prosthesis once it has been implanted during cardiac surgery, which increases the importance of preoperative consultation. It is critical to conduct a comprehensive medical interview to determine if there is any history of hypersensitivity to jewelry, coins, leather goods, cement, stainless steel, paint, and foods such as chocolate, cocoa, beans, spices, shellfish, and embryos that contain metals such as nickel, chromium, and cobalt to confirm the presence of metal allergies. It is also essential to ascertain whether there are symptoms of suspected metal allergy after dental treatment, as in this case. The use of the causative allergen should be avoided if the presence of an allergy is confirmed. As previously stated, clinicians must remember that even patch tests can have false-negative results and treatment should exclude any suspected substance.

We planned to perform AVR using a bioprosthetic valve in this patient. We decided to use the Avalus valve (Medtronic, Minneapolis, MN, USA), which does not contain metal in its stent, since the presence of multiple metal allergies had been confirmed. The Avalus valve stent is made of polyether ether ketone material and contains barium sulfate to facilitate X-ray visualization [11]. The list of metals contained in bioprosthetic valves available in Japan is shown in Table 1. In addition to the Avalus valve, the other available metal-free bioprosthetic valves, such as the Freestyle valve (Medtronic, Minneapolis, MN, USA) and Solo-smart (LivaNova PLC, London, UK), are stentless valves, all of which require complicated implantation procedures. A shorter operative time should also be a consideration for elderly patients requiring implantation of a bioprosthetic valve, and for those with complications such as interstitial pneumonia, as in this case. A simpler procedure is preferable in terms of shortening the operative time, and the Avalus valve, which does not contain metal in the stent, is a very useful prosthetic valve for cases of suspected metal allergy, especially in the elderly and patients with complications, because of its easy implantation technique.

**Table 1:** Metal components of aortic bioprostheses

	Device	Manufacturer	Metallic elements
Stented tissue valve	Avalus	Medtronic	None
	CROWN	LivaNova	W
	Inspiris	Edwards	Co, Cr, Ni, Mo, Mn, Be, Fe
	MAGNA EASE	Edwards	Co, Cr, Ni, Mo, Mn, Be, Fe
	SJM Epic	Abbott	Stentless steel (Ni, Co, Mo, Mn)
	Trifecta GT	Abbott	Ti alloy
Stentless tissue valve	Free style	Medtronic	None
	Solo Smart	LivaNova	None
TAVI valve	Evolute R	Medtronic	Ni/Ti alloy
	Sapien 3	Edwards	Co, Ni, Cr, Mo, Ti, Mn
Sutureless valve	INTUITY Elute	Edwards	Co, Ni, Cr, Mo, Ti, Mn, Be, Fe
	PERCEVAL	LivaNova	Ni/Ti alloy, Co, Cr, W

*Be* beryllium; *Ti* titanium; *Cr* chromium; *Mn* manganese; *Fe* iron; *Co* cobalt; *Ni* nickel; *Mo* molybdenum; *W* tungsten

Moreover, pacemaker wires and sternal closure devices are also used in cardiac surgery, but the pacemaker wires were removed uneventfully on POD 2 in this patient. Titanium-based products are used for sternal closure; however, for biomedical purposes, titanium is generally used in the form of alloys that contain other metals in small concentrations, and are reports of allergies caused by titanium alloys [4]. In this case, we decided to close the sternum using non-absorbable threads and ensured that no skin symptoms or abnormalities occurred at the implantation site by adopting a surgical strategy that was devoid of the implantation of metal-containing substances in the body.

## Conclusion

We performed AVR using the Avalus valve in a patient with severe aortic stenosis with self-reported allergies to multiple metals. The bioprosthetic Avalus valve does not contain metal in the stent portion and is very useful for AVR in patients with suspected metal allergy.
